# Occam's razor or Hickam's dictum? Allergic bronchopulmonary aspergillosis and eosinophilic granulomatosis with polyangiitis*

**DOI:** 10.1136/thoraxjnl-2015-207280

**Published:** 2015-12-23

**Authors:** Scott R Henderson, Anand Shah, Susan J Copley, H Terence Cook, CD Pusey, Alan D Salama, Philip W Ind

**Affiliations:** 1 Department of Respiratory Medicine, Hammersmith Hospital, London, UK; 2 Division of Medicine, Centre for Nephrology, University College London, Royal Free Hospital, London, UK; 3 Radiology Department, Hammersmith Hospital, London, UK; 4 Department of Histopathology, Hammersmith Hospital, London, UK; 5 Imperial College Kidney & Transplant Institute, Hammersmith Hospital, London, UK.

**Keywords:** ANCA Related Vasculitides, Aspergillus Lung Disease, Systemic disease and lungs

**Anand Shah and Scott R Henderson (AS and SRH):** a 72-year-old retired male physician was referred with poorly controlled asthma, necessitating oral glucocorticoid therapy. He had been diagnosed with occupational asthma 9 years earlier with initially good control on an inhaled corticosteroid (ICS). In the preceding year, his symptoms deteriorated with increasing wheeze, dyspnoea and persistent productive cough with 5 mL sputum daily associated with reduced exercise tolerance of 30–50 metres on the flat. He also reported a postnasal drip and allergic rhinitis. Medical history included two nasal polypectomies and paroxysmal tachycardias, with normal 24 h Holter, echocardiogram and exercise tolerance test.

He had previously worked as an occupational physician for 15 years, monitoring coffee bean handlers for asthma and sensitivity to *Aspergillus* spp, commonly found in coffee bean cargoes. Interestingly, he himself was indirectly exposed to coffee bean dust (including green coffee beans prior to roasting and castor bean dust) through his interaction with workers and his place of work. He then developed asthma symptoms 6 years after taking up his Occupational Physician post, consistent with a diagnosis of occupational asthma, and a series of skin prick tests demonstrated sensitivity to *Aspergillus fumigatus*.

Examination at presentation revealed no significant abnormalities. Investigations showed FEV_1_ of 1.5 L (45% predicted), FVC of 3.0 (63% predicted) and peak expiratory flow (PEF) of 280 L/min (predicted). Sputum cytology revealed 12% eosinophils. Total IgE was elevated at 219 (0–120) kU/L with *Aspergillus* radioallergosorbent test (RAST) at 4.05 (0–0.34) IU/mL. Peripheral eosinophil count, on prednisolone, was normal. Skin prick tests were positive to *A. fumigatus*, grass mixture and house dust mite. Chest radiograph was unremarkable.

**Philip W Ind (PWI):** lung function tests demonstrated an obstructive defect with an FEV_1_:FVC ratio of 50% and reduced PEF. In addition to probable IgE-related occupational asthma, his previous allergen exposure, documented immune reactivity to *Aspergillus* spp and failure to respond to conventional therapy raise the possibility of allergic bronchopulmonary aspergillosis (ABPA). The possible differential diagnosis of continued exposure to workplace allergens resulting in worsening of his condition did not apply as he was retired.

He was intolerant of long-acting β2 agonists, theophylline was relatively contraindicated by previous tachycardias and a tapering dose of prednisolone and high-dose ICS may be inadequate to control symptoms. A leukotriene antagonist (LTRA) was tried in view of his nasal symptoms. High resolution CT scan (HRCT) was also arranged to evaluate lung parenchyma.

**Figure 1:**
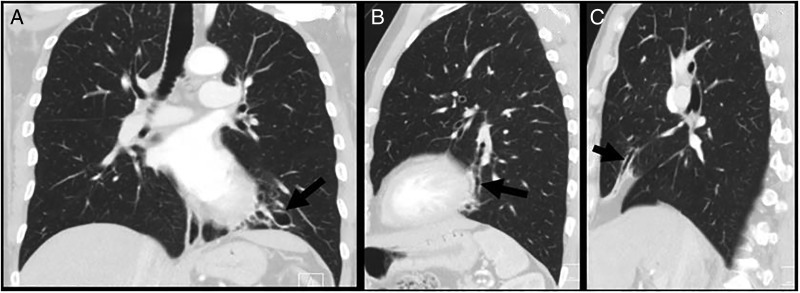
Coronal (A) and sagittal (B and C) CT reconstructions demonstrating right middle lobe (C) and left lower lobe (A and B) bronchiectasis (arrows) consistent with allergic bronchopulmonary aspergillosis.

**Susan J Copley (SJC):** thoracic HRCT demonstrated right middle lobe and left lower lobe bronchiectasis (figure 1) which could be consistent with ABPA.

**AS and SRH:** LTRA therapy was complicated by a severe rash over the legs that resolved on stopping the medication. Tapered prednisolone and high-dose ICS produced improvement. However, 3 months later, he re-presented with a 5-week history of severe muscle cramps, radiating from the buttocks to the lower legs. There was no associated urinary retention and no loss of perianal sensation, but he noted paraesthesia of both hands and feet and drenching night sweats. Neurological examination revealed reduced power (4+/5) in shoulder abduction, hip flexion and hip extension bilaterally, and diminished pinprick sensation over the fourth and fifth fingers in both hands and in a stocking distribution in his legs.

Investigations at this time showed elevated total white cell count of 17.5×10^9^/L with peripheral eosinophilia of 7.1×10^9^/L and elevated C reactive protein of 71 (0–10 mg/L). Multiple sputum samples were negative for bacteria and fungi. Chest X-ray was again normal. MRI of the spine did not reveal any neural compression, but mild degenerative changes. Antineutrophil cytoplasm antibody (ANCA) was positive with perinuclear immunofluorescence staining pattern (p-ANCA) and ELISA demonstrated elevated antimyeloperoxidase (MPO) antibody titre of 107 AU/mL (0–30).

**Alan D Salama (ASD)**: the history of severe myalgia with peripheral neuropathy and elevated inflammatory markers suggests the possibility of a systemic inflammatory process such as vasculitis. Previous history of worsening asthma, nasal polyps, elevated eosinophil count and positive ANCA with a specific anti-MPO antibody is consistent with eosinophilic granulomatosis with polyangiitis (EGPA). It is important to confirm the diagnosis histologically, if possible, and explore the extent of the disease. The patient's urine showed no evidence of blood or protein on dipstick testing. In addition, there were no cells or casts seen on microscopy. With a bland urine sediment, the most useful way to obtain a tissue diagnosis would be sural nerve biopsy.

**Figure 2:**
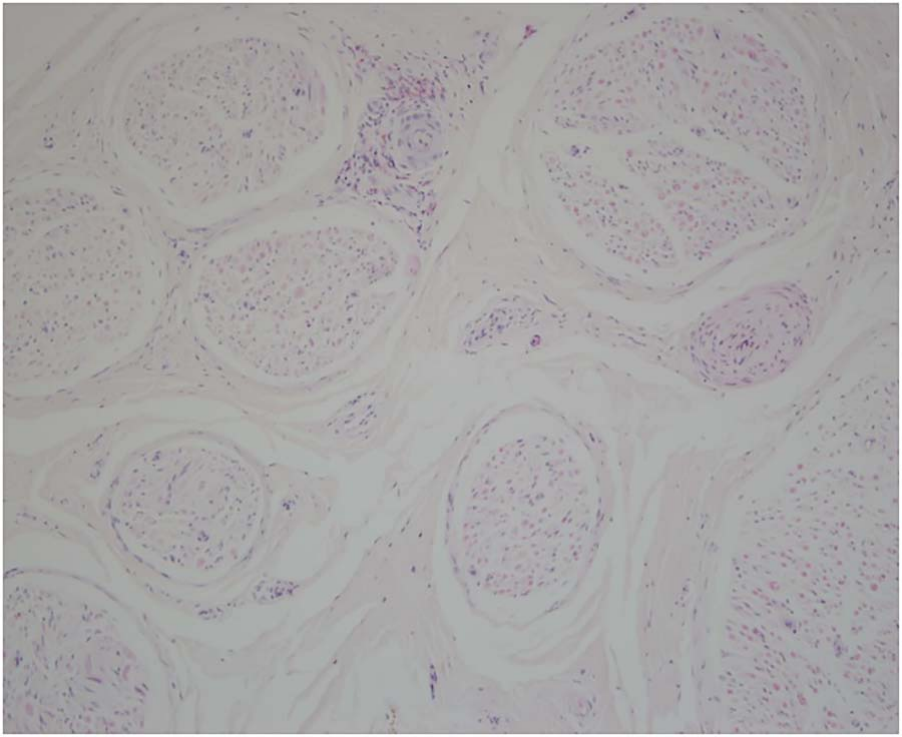
Sural nerve biopsy showing two epineural arteries with fibrosis and luminal narrowing. One of these is surrounded by eosinophils. The appearances are consistent with arterial damage from vasculitis, but there is no active fibrinoid necrosis. The presence of the eosinophils is consistent with eosinophilic granulomatosis with polyangiitis.

**H Terence Cook (HTC)**: sural nerve biopsy (figure 2) shows two epineural arteries with fibrosis and luminal narrowing. One of these is surrounded by eosinophils. The appearances are consistent with arterial vasculitis, but there is no active fibrinoid necrosis in this specimen. The presence of eosinophils is consistent with EGPA.

**PWI**: this patient has evidence of both ABPA and EGPA which may have occurred in relatively quick succession. Immunosuppression is important in managing both these conditions, and the patient should be started on a combination of a glucocorticoid and a steroid sparing agent such as azathioprine.

**AS and SRH**: this patient's clinical symptoms markedly improved following immunosuppression with prednisolone 60 mg and azathioprine 100 mg. Anti-MPO antibody titres and inflammatory markers diminished rapidly and normalised after 2 months’ treatment. He continues to do well with maintenance low-dose prednisolone and azathioprine and high-dose ICS. It is, however, unclear whether his occupational asthma and diagnosis of ABPA and EGPA are pathologically linked.

**PWI**: ABPA represents a severe complication of *A. fumigatus* sensitisation which, in this patient, is likely to be related to prolonged occupational exposure to *Aspergillus* from coffee beans, leading to an IgE-directed immune response resulting in airway and parenchymal lung damage. Premorbid occupational asthma and atopic symptomatology support the patient's increased susceptibility to ABPA, the most common antigen-specific hypersensitivity disorder within the UK. In addition, a latency period can exist between exposure to coffee bean dust, *A. fumigatus* sensitivity and occupational asthma. This would be consistent with his occupational asthma initially occurring 6 years after exposure to the coffee bean plantation, and an additional disease process evolving 9 years later, such as the development of ABPA.

However, the association between ABPA and the emergence of Churg Strauss syndrome (CSS) is extremely unusual. To my knowledge, only two cases of ABPA ‘progressing’ to allergic granulomatosis and angiitis or EGPA have been reported.^1^ ^2^ The precise mechanism(s) by which ABPA and possibly other allergic mycoses could relate to a systemic vasculitis such as EGPA are unclear. Both show evidence of disordered humoral immunity, with IgE elevation in allergic mycoses and presence of ANCA in some patients with EGPA; however, these are generally IgG isotypes. Eosinophilic reactivity is present in both but is most marked in cases of EGPA.

**ASD**: autoantibodies, in particular antibacterial permeability increasing protein, are associated with infective lung disease such as cystic fibrosis and non-cystic fibrosis bronchiectasis. It is noteworthy that ANCA can also be a feature of infective lung disease. In the same way that reducing bacterial load is important, treatment of fungal colonisation/infection with anti-fungals (in addition to glucocorticoids) was considered in this patient with the aim of reducing inflammatory processes resulting in autoantibody production.

Numerous *A. fumigatus* antigens are recognised that stimulate both humoral and cellular immune responses mediated directly and indirectly by allergens binding to IgE/IgG and stimulating CD4+ Th2 cell subsets. Human leucocyte antigen (HLA) association has been found in ABPA, in small cohort studies, with increased frequencies of HLA-DR2 and DR5. Although recent data have also demonstrated genetic risk factors for EGPA, including HLA-DR genes (HLA-DRB4) and interleukin-10 production, the precise trigger initiating EGPA is unknown. Eotaxin-3, an eotactic chemokine (CCL26), has been shown to be a very specific marker in EGPA and may be a useful biomarker for disease monitoring, but whether elevated levels occur in patients with EGPA in response to certain environmental antigens, such as Aspergillus, is unknown.^3^ However, it is of interest to note that eotaxin-3 was shown to be the only eotaxin upregulated in asthmatics following respiratory allergen challenge,^4^ lending support to the notion that certain allergens may provoke production of this chemokine, which, in genetically susceptible individuals, may promote excessive eosinophilic recruitment and damage.

The pathogenic significance of anti-MPO autoantibodies, found in 30%–40% of patients with EGPA at diagnosis, is less established. Presence of anti-MPO antibodies has a closer association with microscopic polyangiitis, presenting with necrotising glomerulonephritis, alveolar haemorrhage, purpura and mononeuritis multiplex. A previous suggestion of cross-reactivity between MPO and eosinophilic peroxidise has not been confirmed. Therefore, there may be common antigen/allergen presentation in genetically susceptible individuals mediating both ABPA and EGPA; however, this association remains uncommon. Of note, we did not measure this patient's eotaxin-3 level or have his HLA-DR typing.

**Charles D Pusey (CDP)**: another possible link is the use of LTRA therapy in the management of ABPA. Numerous cases of EGPA have been reported following the introduction of LTRAs for the management of asthma. Although this association has often been attributed to therapeutic withdrawal or cessation of corticosteroids, unmasking EGPA, an increasing number of cases have since been reported showing a spectrum of EGPA development in the presence and absence of concomitant corticosteroid use. Recent systematic review of this possible iatrogenic association demonstrated a clear temporal relationship between LTRAs and onset of EGPA.^5^ The exact mechanism by which LTRAs may induce EGPA remains unknown, and there is a distinct lack of experimental work to guide understanding. Nonetheless, the occurrence of 63 cases of EGPA on LTRAs, reported to the Committee on Safety of Medicines, over the last 30 years highlights the need for cautious introduction of selective cys-LT1 receptor antagonists in patients with severe asthma.
